# Supplementary material for: Chromothripsis during telomere crisis is independent of NHEJ, and consistent with a replicative origin
Source: Genome Res. 2019 May;29(5):737–49. doi: 10.1101/gr.240705.118 (PMC6499312; doi:10.1101/gr.240705.118)
Supplement: Supplemental Material [file supp_gr.240705.118_Supplemental_file_1.zip › contigs/annotated_contigs/DB46/contig.2.DB46_length_567_mean_cov_5.29100529101.docx]

**DB46_length_567_mean_cov_5.29100529101**

```
TTATTCCGGAACTTTTGAAAACAACCCTTCTACAATGCTGTACTCACAACTAACACAGTTCGTTTTCAAAAGTATAGTAAGGAAAACAA
 >chrX:136704939-136705115 - E=3e-94
CATTTTCTATGTATATAGCTGCAGGAAAAAAACTGAAAATATTACACTGCATGAATACATCACATACCAAAGTTTGCAACCAAA|CTG|
                                                                                     >ch
TCATCCAGGCTAGAGTTCAGTGGCATGATCTCGGCCCACCACAACCTCAGCTCCCAGGCTCAAGCAATCCTCTCACCTCAGCCTCCCAA
rX:136703548-136703942 - E=1e-222
GGAGCTGTGACCACAGGCATGTGCCACCATGCCTGGCTAATTTTTTGTAGAGATGGGGTTTTGCTATGTTGGACAGACTGGTCTTGAAT

TCCTGGGCTCAAGTGATCTGACCACCTCGGCCTCCCAAAATGCTGGAAATACAGGCAGGAGCCACTGCACCCAGCCCCAATCAAATATT

AAATGTTGATGTATTTCTCATTGTATTTACTACTTGAGTCATTCAATAAGATAATATAAGAACATCTTTGGTTTCATATGCCTGATCTA

CGTTAGAATTTTGCAGCTTTGAGAACACTACAAAT
```
